# Degenerate Families

**Published:** 1898-01

**Authors:** 


### DEGENERATE FAMILIES.

There is a paper on "The Causes of Poverty," by the late Francis A. Walker, in the December *Century.* Gen. Walker says:

"The true predominant causes of pauperism, as of crime, have been strikingly and painfully brought out in tracing the history of a few families. Three cases will suffice. The reader remembers the investigation of the Jukes family in New York State. Mr. Dugdale estimated that the members of this family, descendants of one worthless woman or intermarried with her descendants, have in seventy-five years cost the State, as criminals and paupers, a million and a quarter of dollars. The history of a Kentucky family founded in 1790 has been traced to include the character and conduct of a host of its members by descent or by sexual alliance legitimate or illegitimate. Among these have been 121 prostitutes. Thieving and beggary have made up the lives of most of the remainder. Those who try to do something better for themselves prove unable to perform hard labor or to endure severe weather. They break down early and go easily to the poorhouse or the hospital. From Berlin we have the history of another criminal and pauper family, the descendants of two sisters who lived in the last century. Their enumerated posterity numbers 834. Of these the history of 709 has been traced with tolerable accuracy. They embrace 106 illegitimate children, 164 prostitutes, 17 pimps, 142 beggars, 64 inmates of poorhouses, and 76 who have been guilty of serious crimes. Still other instructive cases are given, in one of which nearly all the inmates of a county poorhouse have been found to be related in blood.—*The Sanitarian.*

